# Fournier's Gangrene in a Heterosexual Man: A Complication of *Neisseria meningitidis* Urethritis

**DOI:** 10.1155/2012/312365

**Published:** 2012-12-05

**Authors:** Tariq A. Khemees, Brian S. Porshinsky, Abhishek P. Patel, Christopher D. McClung

**Affiliations:** ^1^Department of Urology, The Ohio State University Wexner Medical Center, Columbus, OH 43212, USA; ^2^Department of Plastic Surgery, The Ohio State University Wexner Medical Center, Columbus, OH 43212, USA

## Abstract

A 55-year-old heterosexual male presented to the emergency department with a symptomatology consistent with urethritis and Fournier's gangrene. Urethral swab and operative tissue cultures were positive for coagulase-negative *Staphylococcus* and an intracellular Gram-negative diplococcus. The latter was initially thought to be *Neisseria gonorrhea*; however, DNA sequencing technique confirmed it to be *Neisseria meningitidis*. The patient required three separate surgical debridements to control widespread necrotizing infection. Following documentation of sterile wound healing with appropriate antibiotics, four reconstructive surgeries were necessary to manage the resultant wound defects. To our knowledge, *Neisseria meningitidis* as a causative organism in Fournier's gangrene has not been reported in the literature.

## 1. Introduction

Fournier's gangrene (FG) is a urologic emergency characterized by rapidly progressive necrotizing fasciitis of the male genitalia skin arising from infections in the perineal skin, scrotum, urethra, or the rectum. Multiple predisposing factors have been identified including diabetes mellitus, local trauma, paraphimosis, and urinary extravasation from urethral strictures [1]. Urologists must maintain high index of suspicion when encountering cellulitis at the genital region. A prompt diagnosis is necessary as infection can advance rapidly and result in a high mortality rate, particularly in diabetics, alcoholics, and in those with colorectal source of infection [2]. Emergent surgical debridement is necessary to control the infectious process, which may inevitably result in tissue loss. Bacterial cultures from FG wounds usually reveal polymicrobial infection that grows both aerobic and anaerobic pathogens such as *Streptococci*, *Staphylococci*, coliforms, *Klebsiella*, clostridia, *Corynebacteria*, and bacteroides [2, 3]. While urethritis caused by *Neisseria meningitidis* is an established clinical entity with frequent occurrences in heterosexual men [4–9], to our knowledge its implication in FG has not been documented. In this study we present the first case of FG that was caused by *Neisseria meningitidis* urethritis in a heterosexual man following orogenital transmission. The clinical surgical management will be described.

## 2. The Case 

### 2.1. Patient Presentation

**Figure 1:**
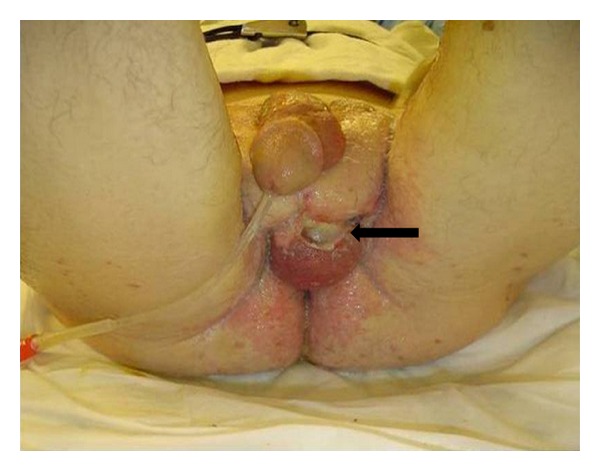
Fournier's gangrene caused by *Neisseria meningitidis*: extensive perineal skin edema, erythema, and necrotic skin loss with exposed left testicle (black arrow).

**Figure 2:**
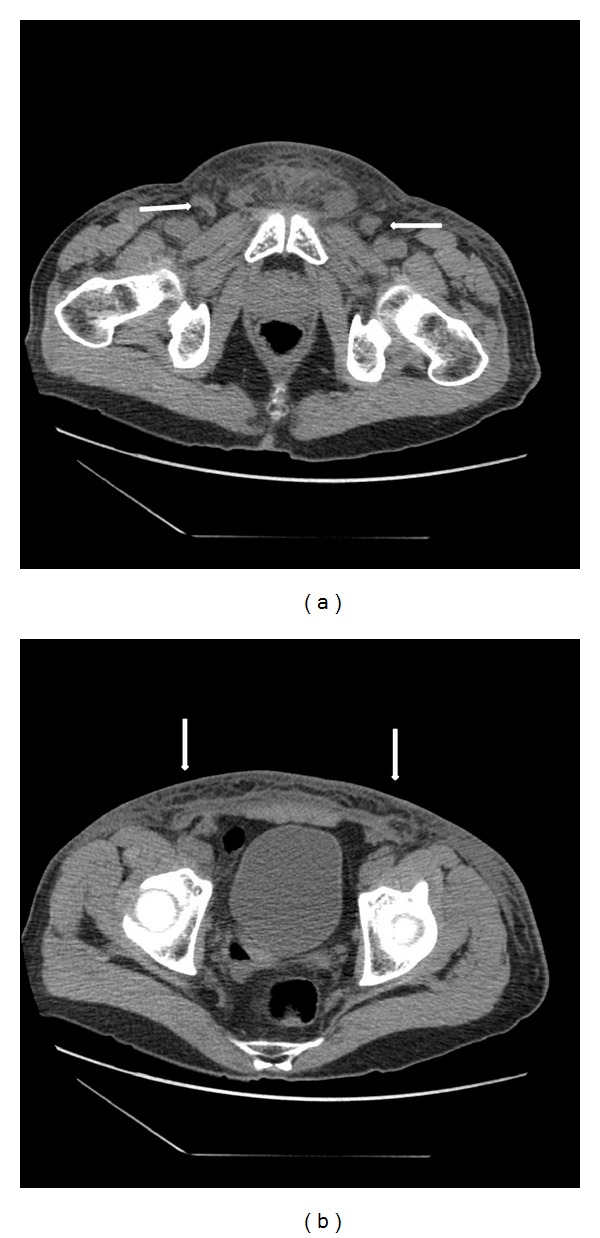
Computed tomography of abdomen and pelvis showing (a) extensive edema and stranding of subcutaneous tissue at the symphysis pubis and bilateral groin areas; bilateral enlargement of inguinal lymph nodes is also noted (two arrows). (b) Edema and fat stranding extending into subcutaneous tissue at anterior lower abdominal wall area (two arrows).

A 55-year-old Caucasian male presented to the emergency department at our institution with the complaint of a two-day history of genital and lower abdominal pain. This was accompanied by increasing genital and lower abdominal skin erythema, along with a foul smell urethral discharge. The patient's sexual history was significant for living with a same-sex partner for 11 years. He also reported an episode of orogenital sexual encounter with another female three months prior to his current presentation. At admission, the patient was hypotensive with a blood pressure of 86/57 and a heart rate of 90 beats per minute. His temperature was 100.7 F. Physical examination revealed diffuse erythema of the skin over the scrotum, penile shaft, and lower abdomen at the symphysis pubis area. There were areas of full-thickness skin necrosis intermixed within the erythematous skin lesions (Figure 1). The affected skin was warm and exquisitely tender to palpation. Purulent urethral discharge was documented, and swab was sent for direct smear and bacterial culture examinations. His laboratory investigations were significant for normal white blood cell count of 4.7 K/uL with 82% of the cells being segmented neutrophils, representing a left shift. CT scan of abdomen and pelvis was performed and revealed subcutaneous tissue stranding in the genital and lower abdominal area with bilaterally enlarged inguinal lymph nodes (Figure 2). Given the suspicion of necrotizing skin infection and underlying urethritis, the patient was started on Vancomycin (1.5 gm/once daily dose, I.V.), *Zosyn* (Piperacillin and Tazobactam) (4.5 gm/three times a day, I.V.), and Doxycycline (100 mg/twice daily, P.O.) for polymicrobial coverage; a prompt surgical debridement was also planned.

### 2.2. Emergent Surgical Management

**Figure 3:**
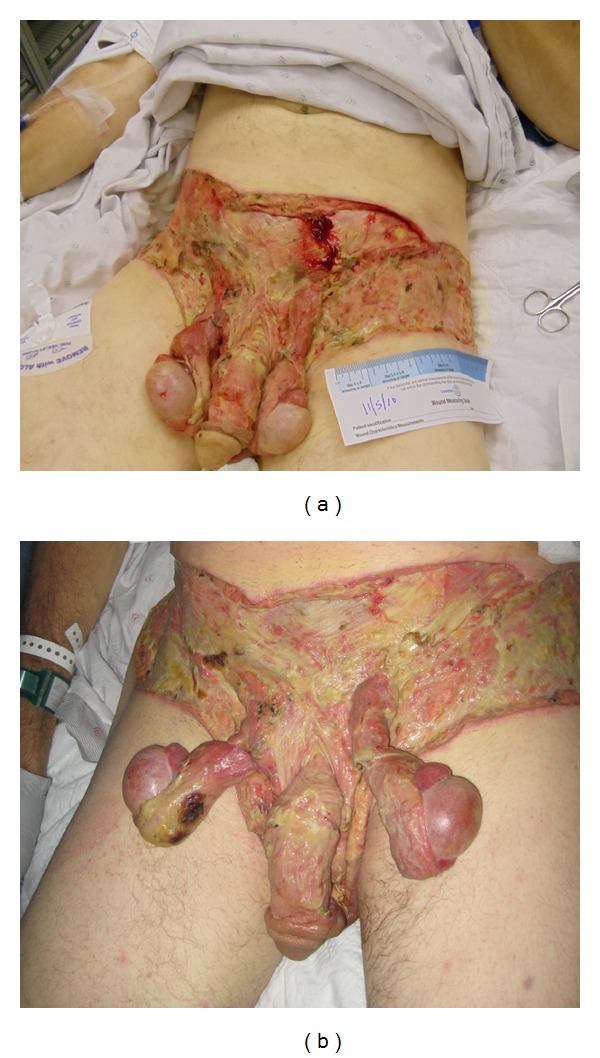
Fournier's gangrene caused by *Neisseria meningitidis*: (a) the patient's wound after three surgical debridements; (b) the same wound following two weeks of daily dressing, vacuum-assisted wound closure, and antibiotic regimen.

The patient was taken emergently to the operating room for exploration. Incision of the affected skin revealed full-thickness necrosis that was also confirmed by histopathological specimen examination. Widespread surgical debridement of his genital and lower abdominal affected skin was required. Within the next four days, the patient required another two wound debridements to control the necrotizing infection (Figure 3(a)). The patient's urethral swab and cultures from the debridement tissues were positive for coagulase-negative *Staphylococcus* and an intracellular Gram-negative diplococcus. The latter was initially thought to be *Neisseria gonorrhea*. On admission day 5, his culture diagnosis was confirmed to be *Neisseria meningitidis* identified using DNA sequencing technique. Anaerobic and fungal cultures did not grow any pathogen. The patient was consented for HIV testing that was negative. His antibiotic regimen was modified from Vancomycin and *Zosyn* to Ceftriaxone (single dose of 125 mg, I.M.), Vancomycin (1.5 gm/once daily dose, I.V.), and oral Azithromycin (single dose 1200 mg) to provide coverage for nogonococcal urethritis. A vacuum-assisted wound closure and cadaveric skin were utilized to accelerate healing. Following completion of two weeks on this regimen, the necrotizing infection was controlled, and negative wound cultures were documented (Figure 3(b)). Subsequently, the patient's first reconstructive procedure was planned.

### 2.3. Reconstruction of Fournier's Gangrene Wounds

**Figure 4:**
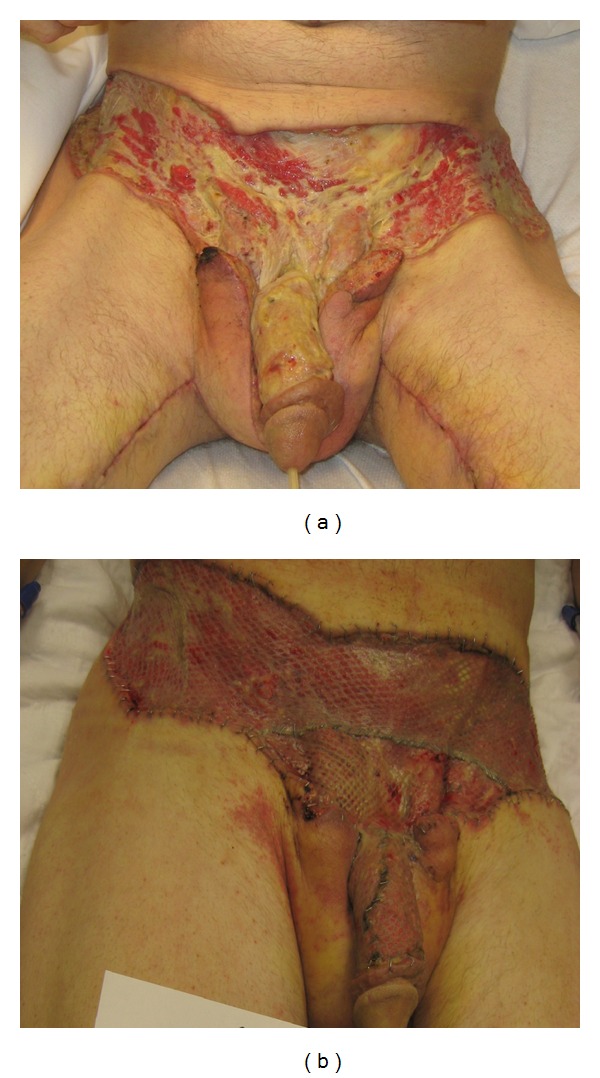
Reconstruction of Fournier's gangrene wound: (a) bilateral medial thigh fasciocutaneous flaps for scrotal reconstruction were performed; (b) cadaveric skin graft of the penis and lower abdomen was necessary for temporary wound cover.

Bilateral medial fasciocutaneous thigh flaps were performed for scrotal reconstruction. Each flap was 15 cm long with a base of 5 cm, and both were transposed and adequately reached to cover the defect. The tips of the two flaps were sutured together creating a midline raphe. The flaps provided adequate coverage of the testes with a cosmetically acceptable neoscrotum. Both flaps and donor sites healed nicely without complication (Figure 4(a)).

**Figure 5:**
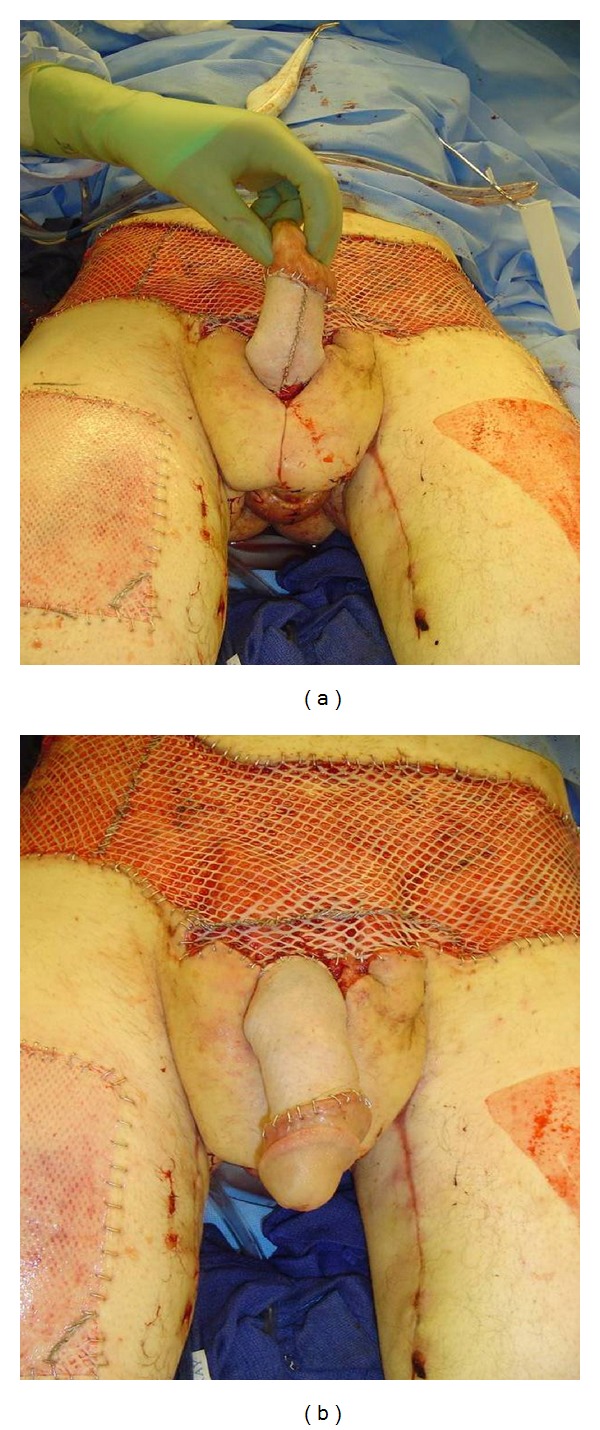
Full-thickness skin graft with 3 : 1 mesh was used to cover lower abdominal wound, while a thick-split-thickness skin graft was used to cover penile shaft skin loss. Allograft skin was obtained from the patient's thighs.

**Figure 6:**
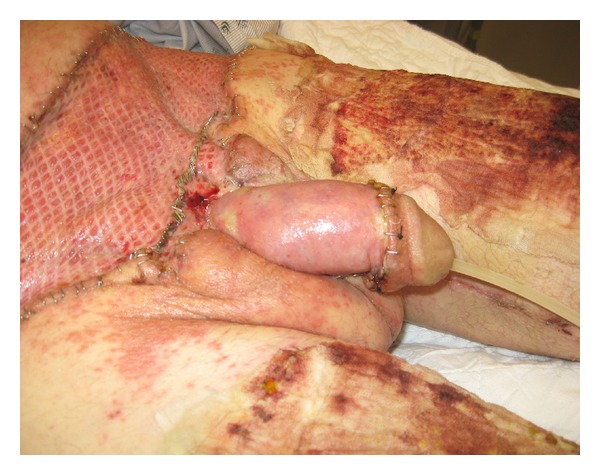
Healing of Fournier's gangrene wounds: skin graft and donor sites one week after surgery.

Attention was then shifted toward reconstructing the patient's large abdominal and penile skin defect. Cadaveric skin grafts were used to temporarily cover penile and lower abdominal defects (Figure 4(b)). Three weeks later, the cadaveric skin graft was removed, and a split thickness skin graft with 3 : 1 mesh was utilized to cover 501 cm^2^ lower abdominal wound defect. A thick split-thickness skin graft (Zimmer dermatome set at 0.024 of an inch) was used to cover the 88 cm^2^ penile skin defect (Figure 5). All skin grafts were obtained from the patient's thigh area. The patient had 100% skin grafts take, and both medial thigh flaps remained viable (Figure 6). Six months following the procedures, the patient developed a ventral penile contracture scar causing chordee during erection. This was managed successfully with scar tissue excision and full-thickness skin graft of the ventral penile shaft.

At time of this report, the patient's wounds have completely recovered, and all donor sites were well healed. The patient states that he has good erectile function of normal length. Written informed consent was obtained for publication of this paper and accompanying images.

## 3. Discussion

FG is a rare but rapidly progressive male genital skin infection that demands early recognition, aggressive treatment with an emergent surgical debridement, and broad-spectrum antibiotic regimen to reduce patient's morbidity and mortality [2]. Mixed bacterial culture is common in FG [10]. In our patient, bacterial cultures grew two different pathogenic species: coagulase-negative *Staphylococcus* and *Neisseria meningitidis*. While coagulase-negative *Staphylococci* were originally reported in the literature as harmless skin commensals and frequently dismissed as culture contaminants, their important role as pathogenic bacteria causing human illnesses is now well documented [11, 12]. Additionally, previous reports have shown that this organism may frequently be the cause of necrotizing fasciitis and FG [13–15].

*Neisseria meningitidis* is a human commensal that resides in the nasopharynx as its main reservoir. The bacterium can transform into a pathogenic strain causing invasive disease in humans. High genetic variability that leads to a continued change in virulence and transmissibility has been reported [16]. Based on immunospecificity of the meningococcal capsule, five serogroups of pathogenic *Neisseria meningitidis* have been linked to a spectrum of illnesses including meningitis, pharyngitis, pneumonia, pericarditis, arthritic syndrome, prosthetic joint infections, and urethritis [17]. Urethritis caused by *Neisseria meningitidis* is relatively uncommon, but the incidence of the disease is reportedly increasing over the past years secondary to changes in sexual behavior [8, 18, 19]. Homosexual men have encountered a higher disease prevalence. Given that the nasopharynx is the favored reservoir for the bacterium, orogenital contact has been proposed as a probable route of transmission in heterosexual men [4–7]. This mode of contact has been confirmed by the work of Urra et al. who showed an association between the genotypes of causative pathogen of urethritis in a heterosexual man and his sexual partner using pulsed-field gel electrophoresis [9].

The mortality rate of Fournier's gangrene has been decreasing in modern times. In a recent population-based study, the overall mortality rate was cited to be 7.5% [20]. Aggressive surgical management has been traditionally linked to improved survival in FG [21, 22]. Management of wounds after debridement and subsequent reconstruction can be challenging. In the present case, postoperative wound management was aided by the use of vacuum-assisted dressing and cadaveric skin. Multiple reconstructive surgeries were also required, but the functional and cosmetic outcomes were excellent.

## 4. Conclusion

To our knowledge, this is the first paper to document *Neisseria meningitidis* as a causative factor of Fournier's gangrene. In our patient, urethritis caused by this organism led to the development of Fournier's gangrene that was treated with appropriate surgical debridement and proper antibiotic. This pathogen should be on the differential as an atypical causative agent for the disease, and proper diagnostic testing should be applied in FG patients presenting with urethritis.
